# Enhanced production of styrene by engineered *Escherichia coli* and in situ product recovery (ISPR) with an organic solvent

**DOI:** 10.1186/s12934-019-1129-6

**Published:** 2019-05-03

**Authors:** Kyungsoo Lee, Hyun Bae Bang, Yoon Hyeok Lee, Ki Jun Jeong

**Affiliations:** 1 Department of Chemical and Biomolecular Engineering (BK21 Program), KAIST, 291 Daehak-ro, Yuseong-gu, Daejeon, 34141 Republic of Korea; 2 Department of Bio and Brain Engineering, KAIST, 291 Daehak-ro, Yuseong-gu, Daejeon, 34141 Republic of Korea; 3 Institute for the BioCentury, KAIST, 291 Daehak-ro, Yuseong-gu, Daejeon, 34141 Republic of Korea

**Keywords:** *Escherichia coli*, Styrene, Fed-batch culture, In situ product recovery (ISPR)

## Abstract

**Background:**

Styrene is a large-volume commodity petrochemical, which has been used in a wide range of polymer industry as the main building block for the construction of various functional polymers. Despite many efforts to produce styrene in microbial hosts, the production titers are still low and are not enough to meet the commercial production of styrene.

**Results:**

Previously, we developed a high l-phenylalanine producer (*E. coli* YHP05), and it was used as a main host for de novo synthesis of styrene. First, we introduced the co-expression system of phenylalanine-ammonia lyase (*PAL*) and ferulic acid decarboxylase (*FDC*) genes for the synthesis of styrene from l-phenylalanine. Then, to minimize cell toxicity and enhance the recovery of styrene, in situ product recovery (ISPR) with *n*-dodecane was employed, and culture medium with supplementation of complex sources was also optimized. As a result, 1.7 ± 0.1 g/L of styrene was produced in the flask cultures. Finally, fed-batch cultivations were performed in lab-scale bioreactor, and to minimize the loss of volatile styrene during the cultivation, three consecutive bottles containing n-dodecane were connected to the air outlet of bioreactor for gas-stripping. To conclude, the total titer of styrene was as high as 5.3 ± 0.2 g/L, which could be obtained at 60 h.

**Conclusion:**

We successfully engineered *E. coli* strain for the de novo production of styrene in both flask and fed-batch cultivation, and could achieve the highest titer for styrene in bacterial hosts reported till date. We believe that our efforts in strain engineering and ISPR strategy with organic solvent will provide a new insight for economic and industrial production of styrene in a biological platform.

**Electronic supplementary material:**

The online version of this article (10.1186/s12934-019-1129-6) contains supplementary material, which is available to authorized users.

## Background

Styrene is a large-volume commodity petrochemical whose global production exceeded 26.4 million tons in 2012 [1]. Particularly, styrene has been used in a wide range of polymer industry as it is the main building block for the construction of various functional polymers including styrene butadiene rubber (SBR), expanded polystyrene (EPS), and acrylonitrile butadiene styrene (ABS). Since these functional co-polymers are commonly used in construction fields as insulating materials, consistent increase in demand for styrene production is expected in the near future [2, 3]. The conventional production of styrene solely relies on the petrochemical industry, where petroleum-derived ethylbenzene is converted into styrene through a catalytic dehydrogenation [4]. However, such typical process requires extensive thermal energy of more than 200 trillion BTU of steam annually in the US alone [5]. Therefore, the chemical synthesis of styrene has been regarded as one of the most energy-intensive process among various petrochemical synthesis.

As an alternative, a biological approach was suggested to provide an eco-friendly and sustainable platform for styrene production [6–9]. An economic evaluation revealed that the bio-based styrene production could be competitive with the current petroleum-based platforms [10]. The natural production of styrene has been found in various hosts including microorganisms, such as *Penicillium camemberti* and certain specific plant species [6, 11]. However, their extremely low productivities suggested the need for a suitable host for an economically feasible production. Instead of natural producers, several recombinant hosts including *Escherichia coli* and *Saccharomyces cerevisiae* have been engineered for the production of styrene. McKenna et al. [6] were successful in incorporating phenylalanine-ammonia lyase (PAL) from *Arabidopsis thaliana* and ferulic acid decarboxylase (FDC) from *S. cerevisiae* into a high l-phenylalanine producer strain (*E. coli* NST74) to demonstrate a styrene biosynthesis pathway in *E. coli*, where 260 mg/L of styrene was produced in flask cultures. The production titer of styrene was further enhanced to 836 mg/L in flasks by adopting a two-phase partitioning method using organic solvents [8]. Meanwhile, Liu et al. [9] optimized the biosynthesis pathway to l-phenylalanine in *E. coli* BL21(DE3) based on enzyme screening and a metabolic flux analysis, in combination with in situ product removal (ISPR), resulting in a styrene titer of 350 mg/L of styrene production in flask cultivation. Same pathway has been incorporated into *S. cerevisiae* to produce styrene, where *PAL* and *FDC* genes were overexpressed along with the down-regulation of a competing pathway [7]. However, the titer reached only up to a maximum of 29 mg/L in flask cultivation. Despite these efforts to increase the production titer of styrene in microbial hosts, the current titers are not high enough for the commercial production and it is necessary to develop more potential host and efficient bioprocess.

**Fig. 1:**
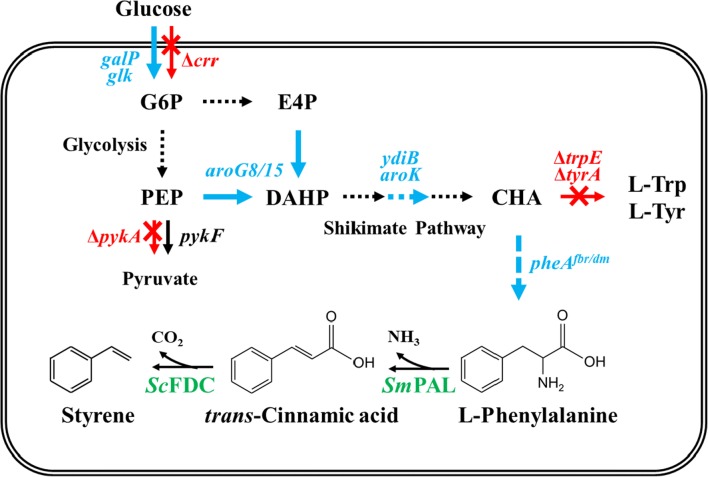
Graphical diagram of the biosynthesis pathway of styrene from glucose. Blue, red and green arrows indicate the overexpression of endogenous gene, the deleted genes, the overexpression of heterologous genes, respectively. G6P: glucose 6-phosphate; E4P: erythrose 4-phosphate; PEP: phosphoenolpyruvate; DAHP: 3-deoxy-d-arabinoheptulosonate 7-phosphate; CHA: chorismate

Previously, we successfully developed *E. coli* that produces *trans*-cinnamic acid (*t*CA) and cinnamaldehyde [12, 13]. In those works, A biosynthesis pathway of l-phenylalanine from glucose in *E. coli* was thoroughly reconstructed to increase the titer of l-phenylalanine, which is the main precursor of *t*CA (Fig. 1). Thus, by introducing phenylalanine-ammonia lyase (*Sm*PAL) from *Streptomyces maritimus* into this high l-phenylalanine producer (*E. coli* YHP05 harboring pYHP and pHB-CA), the production of *t*CA as high as 6.9 g/L was successfully demonstrated [13]. Here, we sought to apply this high l-phenylalanine producer for the production of styrene. First, we constructed a single-step pathway for de novo synthesis of styrene from *t*CA. Next, we tried to enhance the productivity of styrene by optimizing the enzyme expression and culture media. In addition, for minimizing cell toxicity and enhance the recovery of styrene, in situ product recovery (ISPR) strategy with an organic solvent (*n*-dodecane) was employed together. Finally, we performed the fed-batch cultivation with the engineered strain in the lab-scale (5 L) bioreactor to demonstrate the large-scale production of styrene.

## Results and discussion

### Construction of styrene biosynthesis pathway in *E. coli*

**Fig. 2:**
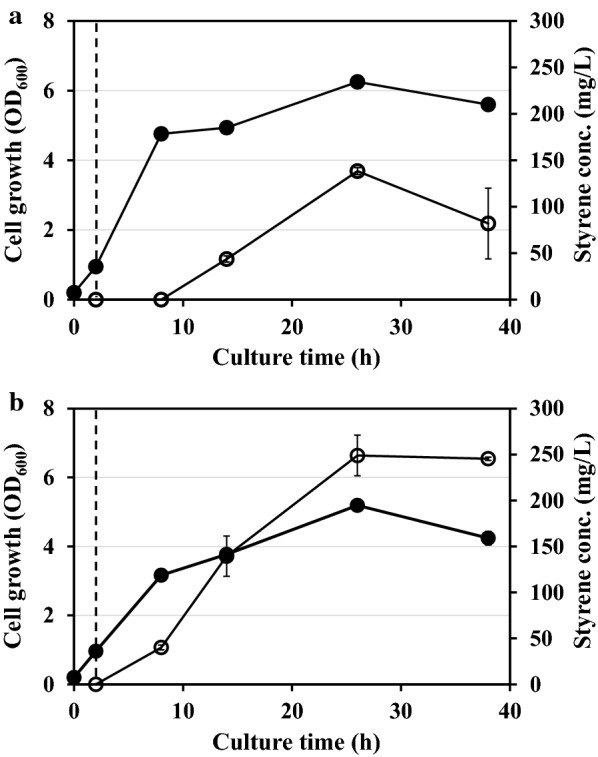
Time profiles of cell density (OD_600_) and styrene production titer in flask culture. **a** *E. coli* YHP05 harboring pYHP and pHB-CA-FDC and **b** *E. coli* YHP05 harboring pYHP-FDC and pHB-CA. Closed and open circles represent cell density (OD_600_) and concentration of styrene, respectively. Dashed line indicates the induction time. The results are shown as the mean value ± standard deviation (SD) of two independent experiments

In *E. coli*, styrene can be synthesized from l-phenylalanine catalyzed by two enzymes: phenylalanine-ammonia lyase (PAL) and ferulic acid decarboxylase (FDC) (Fig. 1). As described earlier, we previously engineered *E. coli* for the enhanced production of *t*CA [12, 13], and to produce styrene, we decided to use this host (*E. coli* YHP05 harboring pYHP and pHB-CA). For the biosynthesis of styrene from *t*CA, we first introduced an *FDC* gene from *S. cerevisiae* (without the signal peptide) into downstream of a *Sm*PAL gene in pHB-CA, yielding pHB-CA-FDC, in which both *Sm*PAL and *Sc*FDC genes were expressed under isopropyl-β-d-thiogalactopyranoside (IPTG)-inducible P_trc_ promoter (Additional file 1: Fig. S1a). To determine the production of styrene, *E. coli* harboring pHB-CA-FDC and pYHP was cultivated in Luria–Bertani (LB) medium. It is known that styrene titers higher than 0.2 g/L give an inhibitory effect on the growth of *E. coli* [9]. Therefore, to alleviate the inhibitory effect of styrene, we used ISPR method with *n*-dodecane, which was added in the media to extract the styrene produced during the cultivation. In this cultivation, it was clearly confirmed that styrene was successfully produced, and its production titer reached up to 138.3 ± 3.6 mg/L at 26 h (Fig. 2a). When we analyzed the expression levels of both *Sc*FDC and *Sm*PAL genes by SDS-PAGE, it was confirmed that *Sc*FDC gene was successfully expressed after induction (Additional file 2: Fig. S2a). However, we also found that the expression level of *Sm*PAL gene substantially decreased in pHB-CA-FDC compared to that in pHB-CA, where only *Sm*PAL gene was expressed. In the bicistronic expression system such as pHB-CA-FDC, the expression level of the 1st gene can be changed (increased or decreased) by the insertion of 2nd gene [14, 15]. By the insertion of the 2nd gene, the length of mRNA transcript is increased and the elongated mRNA makes mRNA unstable and degraded, so the expression level of 1st gene can be decreased by short half-life of mRNA transcripts. Also, the sequence of 2nd gene (particularly translation initiation region), can give negative effect on the translation of the first gene by the formation of the unfavorable secondary structure. We don’t know the exact reason for the decrease of *Sm*PAL gene in pHB-CA-FDC, but it was necessary to restore the expression level of *Sm*PAL for the enough supplementation of *t*CA.

### Optimization of the enzyme expression levels

Together with *Sc*FDC, the expression of *Sm*PAL gene is highly critical in the production of styrene, and both gene expression levels should be optimized. We noted that *t*CA barely remained in supernatant (data not shown), suggesting that *t*CA has been converted to styrene immediately after it was synthesized from l-phenylalanine by *Sm*PAL-mediated reaction. This result implied that the production of styrene can be further increased by more supplementation of *t*CA and it is necessary to increase the expression level of *Sm*PAL gene. For this purpose, we decided to use the original plasmid (pHC-CA) instead of pHC-CA-FDC, and *Sc*FDC gene was moved to the other plasmid (pYHP), yielding pYHP-FDC in which gene expression was under a constitutive promoter (BBa_J23100) (Additional file 1: Fig. S1b). *E. coli* YHP05 harboring pHB-CA and pYHP-FDC was cultivated in LB medium, and the expression levels of both genes were analyzed by SDS-PAGE. We found that the expression of *Sm*PAL gene in pHB-CA increased and maintained sufficient level (Additional file 2: Fig. S2b). In contrast, the expression level of *Sc*FDC in pYHP-FDC decreased slightly compared to the previous construct (pHC-CA-FDC) due to the plasmid copy number: The backbone of pYHP-FDC is a pTac15K, which has lower copy number than pHC-CA-FDC [13]. In flask cultivation with this strain, the styrene titer was 248.9 ± 22.1 mg/L, which was approximately 2-fold higher compared with the previous system (Fig. 2b). In this cultivation, cells showed a little decrease of cell density (OD of 5.3) compared with that of cell harboring pYHP and pHB-CA-FDC (OD of 6.2), but higher expression of biosynthesis genes (Additional file 2: Fig. S2) could drive more supplementation of main precursor (*t*CA) and consequently more production of styrene.

### Optimization of culture media

**Fig. 3:**
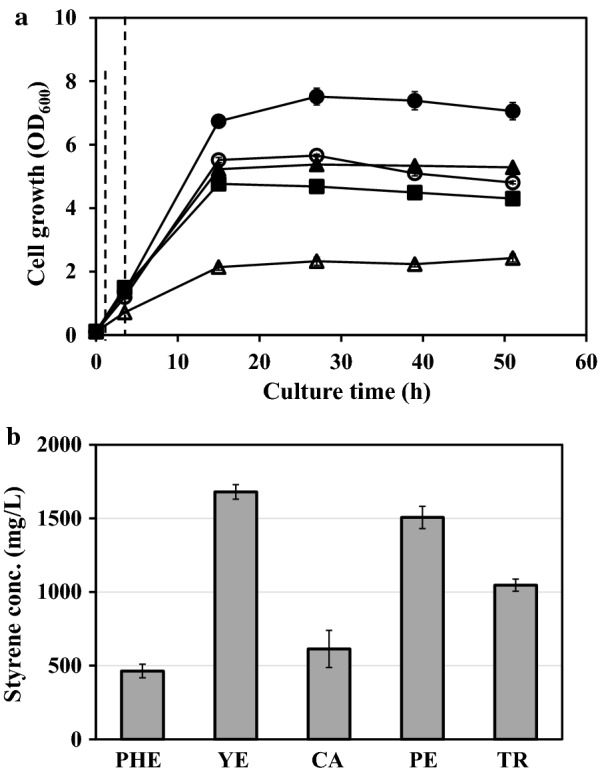
Flask cultivations of *E. coli* YHP05 harboring pYHP-FDC and pHB-CA in defined PHE media supplied with various complex sources. **a** Time profiles of cell density (OD_600_). Dashed line indicates the induction time point. **b** Styrene production titers. Open triangle, no complex source (PHE); closed circle, yeast extract (YE); open circle, casamino acid (CA); closed square, peptone (PE); closed triangle, tryptone (TR). The results are shown as the mean value ± standard deviation (SD) of two independent experiments

Next, we attempted to optimize the media as a cost-effective minimal media instead of complex media. Through our previous efforts on increasing *t*CA production, PHE minimal medium has been developed [12, 13] and was used for the styrene production. *E. coli* YHP05 harboring pHB-CA and pYHP-FDC was cultivated in the PHE medium, and cell growth and the production titer were compared with those in LB complex medium. Without any complex source in PHE medium, cells showed much slower cell growth, and maximum cell density (OD_600_ of 2.4) was also lower compared with those in LB medium (Fig. 3a). However, the production titer of styrene at 48 h was 463.3 mg/L, which was 1.9-fold higher than that in LB medium (Fig. 3b). In minimal media, a few complex sources have been supplied for the increase of cell growth and production, and in these semi-defined media, the choice of complex source for supplementation is also critical for the production yields of target products [16, 17]. To find the best complex source on styrene production, four different complex sources including yeast extract, peptone, tryptone, and casamino acid were examined in the culture media, which are useful for the supplementation of amino acids and beneficial for protein synthesis in *E. coli* [13, 18]. Each complex source was added into the PHE medium as the final concentration of 3 g/L, and cell growth and styrene production were compared. As shown in Fig. 3, cells showed higher cell density and styrene production under the supplementation of complex source than that in PHE medium without any complex source. Among four complex sources, the supplementation of yeast extract showed the most positive effect on both cell growth and styrene production, where optical density at 600 nm (OD_600_) reached 7.5 ± 0.3 and styrene titer reached a maximum of 1.7 ± 0.1 g/L (Fig. 3). Compared with cultivation in LB medium, the production titer was 6.9-fold higher and, to the best of our knowledge, this is the highest styrene production titer in flask cultivation. Among four examined nutrients, tryptone, peptone and casamino acids which are derived by enzymatic digestion or acid hydrolysis of casein or polypeptides, can supply various amino acids which are beneficial for protein synthesis. Yeast extract is also good resource for the supplementation of various amino acids, but it also provides other useful resources including nitrogenous compounds, trace nutrients, vitamin B complex and other important growth factors, which are essential for the cell growth as well as protein synthesis. Although we don’t know the exact contribution of each complex nutrient on styrene production, we suppose that the supplementation of yeast extract containing more various nutrients than others might be more beneficial for cell growth, and higher production of styrene could be achieved through higher cell growth.

In addition to culture media, we also examined the effect of culture temperatures on styrene production. In the previous work for production of *t*CA [12, 13], we examined two temperatures 30 °C and 37 °C, and cultivation at high temperature gave higher production of *t*CA. *E. coli* YHP05 harboring pHB-CA and pYHP-FDC was cultivated in the PHE medium with supplementation of yeast extract at 37 °C, and after induction, cells were cultivated at both temperatures (30 °C or 37 °C). Similar as previous results [12, 13], we confirmed that styrene production was higher by cultivation at 37 °C than 30 °C (Additional file 3: Fig. S3). Those conditions (37 °C and supplementation of yeast extract) was employed in the following fed-batch cultivations.

### Fed-batch cultivation for styrene production

**Fig. 4:**
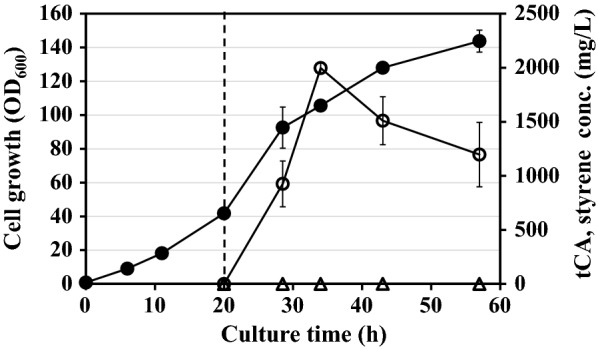
Time profiles of cell density (OD_600_) and styrene production titers in fed-batch cultivation supplemented with n-dodecane. Closed circle, cell density (OD_600_); open circle, styrene. Dashed line indicates the induction time point. The results are shown as the mean value ± standard deviation (SD) of two independent experiments

**Fig. 5:**
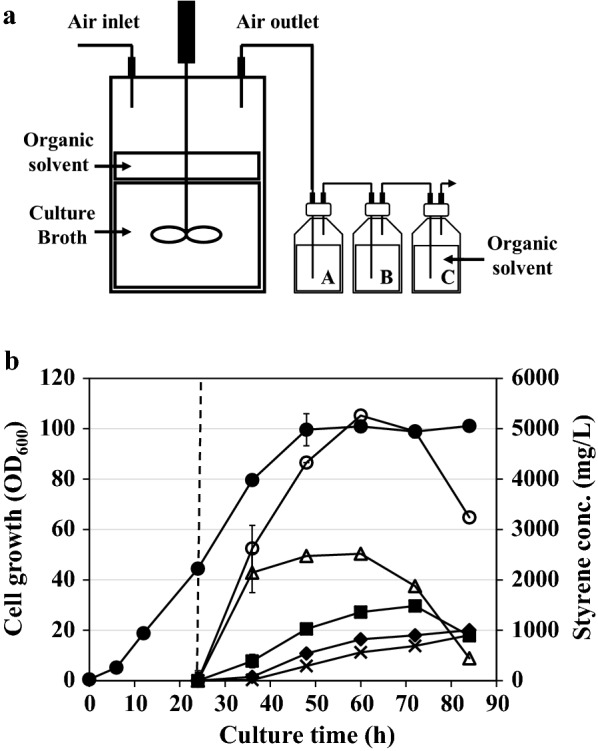
Fed-batch cultivations equipped with exterior extraction modules. **a** Graphical diagram of the exterior extraction modules (three bottles) in fed-batch cultivation. **b** Time profiles of cell density (OD_600_) and styrene production titer. Closed circle, cell density (OD_600_); open circle, total styrene; open triangle, styrene from interior *n*-dodecane; closed square, styrene from reservoir A; closed diamond styrene from reservoir B; cross, styrene from reservoir C. Dashed line indicates the induction time point. The results are shown as the mean value ± standard deviation (SD) of two independent experiments

Next, fed-batch cultivations were performed to examine the performance of the engineered strain for the production of styrene in the lab-scale bioreactor (5 L). Similar to flask cultivation, we also employed ISPR method using an organic solvent (*n*-dodecane). In this fed-batch cultivation, cells were induced at an OD_600_ of 45 for the gene expression, and cells continued to grow up to an OD_600_ of 143.8 (Fig. 4). Immediately after IPTG induction, styrene began to be produced, and the titer of styrene reached its maximum of 2.0 g/L at 34 h (Fig. 4). Compared with the titer in flask cultivation (1.7 g/L), the final titer was only 1.2-fold higher which was not much higher considering the increase in cell density (~ 19-fold). During the fed-batch cultivation, the expression levels of both genes (*Sc*FDC and *Sm*PAL) were also analyzed by SDS-PAGE, but we could not find any significant decrease in the expression level (Additional file 4: Fig. S4). Although *n*-dodecane was supplied to extract styrene in the medium, we reasoned that significant portion of styrene produced in the bioreactor has been stripped out due to the vigorous agitation and constant aeration. Since styrene is highly volatile, loss of significant amount of styrene synthesized may occur through the air outlet line during the fed-batch cultivation in bioreactor, in which the styrene titer can be underestimated. McKenna et al. employed a gas-stripping method in which off-gas styrene was quantified and added to the total production titer of styrene (561 mg/L) [8]. Thus, to minimize the loss of styrene in the off-gas during the fed-batch cultivation, three consecutive bottles, each containing 200 mL of *n*-dodecane, were connected to the air outlet of the bioreactor (Fig. 5a), so that evaporated styrene could be captured inside series of *n*-dodecane organic solvent. After induction at an OD_600_ of 45, cells continued to grow up to an OD_600_ of 100 at 48 h, and then similar cell density was maintained until the end of cultivation (Fig. 5b). The titer of styrene also increased immediately after induction. Inside the bioreactor, the highest titer (2.5 ± 0.1 g/L) was obtained at 60 h and then it gradually decreased. The titers in three exterior bottles (A, B, and C) increased continuously in the post-induction period, although their titers were lower than that in the interior. At 60 h, the total styrene titer reached its maximum value of 5.3 ± 0.2 g/L with a productivity of 88.3 mg/L/h. In addition, we found that styrene concentration in the last bottle (bottle C) continued to increase till the end of cultivation (Fig. 5b), which indicated that styrene was still lost during the process. To minimize this loss further, we need to employ more efficient recovery system in the off-gas: for example, linking of stripping system with cooling jacket condenser and optimization of the air flow rate in air outline [19, 20].

## Conclusions

During the development of styrene production process, we focused on two points: (i) cell engineering for the enhanced production of styrene and (ii) optimization of bioprocess to minimize the loss and toxicity of styrene. To increase the styrene production titer, we employed *E. coli* YHP05 and the introduction of co-expression system of *Sc*FDC gene enabled *E. coli* to produce styrene successfully with high production titers. By combining ISPR, we could achieve the production of styrene as high as 1.7 g/L (in shake flask) and 5.3 g/L (in fed-batch cultivation). To the best of our knowledge, these are the highest titers produced by de novo synthesis of styrene in microbial hosts till date (Table 1). This is a very successful application of *E. coli* YHP05 strain which was previously developed for the overproduction of l-phenylalanine [12], and we believe that this strain can also be a potential host for the overproduction of l-phenylalanine-derived aromatic compounds such as coumaric acid, benzoic acid, pinocembrin, etc. [21]. As described earlier, the titer value of styrene higher than 0.2 g/L gives an inhibitory effect on the *E. coli* cell growth. However, the use of ISPR with *n*-dodecane could eliminate this inhibitory effect and cells could produce a much higher concentration of styrene than the inhibitory concentration (0.2 g/L). In addition, to maximize the recovery yield, three exterior modules containing *n*-dodecane were connected in series, and styrene concentration as high as 2.8 g/L could be recovered from exterior modules, which was higher than that those recovered from bioreactor (2.5 g/L) (Fig. 5). Although the current solvent recovery process needs to be improved further, we believe our efforts in strain engineering and ISPR strategies (solvent extraction and gas stripping) provide new insight for economic and industrial bio-based styrene production.

**Table 1:** Overview on de novo production of styrene in bacterial hosts

Strain	Culture mode	Solvent for extraction	Max. titers (mg/L)	Productivity (mg/L/hr)	Refs.
*E. coli* YHP05	Flask	*n*-Dodecane	1700	35.4	This work
	Fed-batch fermentation	*n*-Dodecane	5300	88.3	This work
*E. coli* NST74	Flask	–	260	9.0	[6]
*E. coli* NST74	Flask	bis(2-Ethylhexyl)phthalate	836	17.4	[8]
*E. coli* BL21(DE3)	Flask	Isopropyl myristate	350	7.3	[9]
*S. cerevisiae*	Flask	–	29	0.6	[7]

## Materials and methods

### Bacterial strains and plasmids

All strains and plasmids used in this study are listed in Table 2. *E. coli* XL1-Blue was used for cloning and plasmid maintenance. *E. coli* YHP05, which was previously engineered to produce *t*CA [12, 13], was used as the main host for styrene production. Regarding the expression of *Sc*FDC gene, it was cloned into pHB-CA and pYHP. Initially, *Sc*FDC gene was amplified from chromosomal DNA of *S. cerevisiae* using polymerase chain reaction (PCR) with FDC-F and FDC-R primers. PCR product was digested with *Eco*RI and *Xba*I, and ligated with pHB-CA, yielding pHB-CA-FDC in which *Sc*FDC gene was located in the downstream of *Sm*PAL gene under P_trc_ promoter. Next, *Sc*FDC gene was amplified using pHB-CA-FDC as a template by PCR with Gibson-F, Gibson-R1, and Gibson-R2 primers. pYHP was digested with *Not*I and they were assembled with the PCR product by Gibson assembly method [22], yielding pYHP-FDC, in which *Sc*FDC gene was expressed under constitutive BBa_J23100 promoter [23]. All DNA manipulations, restriction enzyme digestions, ligations, and transformations were performed according to the standard protocols [24]. All restriction enzymes were purchased from Enzynomics™ (Daejeon, Republic of Korea). PCR was performed using a C1000™ Thermal Cycler (Bio-Rad, Richmond, CA, USA) with PrimeStar™ HS polymerase (Takara Bio, Shiga, Japan). All primers used in this work are listed in Table 3.

**Table 2:** Bacterial strains and plasmids used in this study

	Description	References
Strain		
XL1-Blue	recA1 endA1 gyrA96 thi-1 hsdR17 supE44 relA1 lac [F′ proAB lacI^q^Z ΔM15 Tn10 (Tet^r^)]	Stratagene^a^
W3110	F^−^ l^−^ rph-1 INV(rrnD, rrnE)	Lab stock
YHP05	W3110 Δcrr ΔtyrR ΔtrpE ΔtyrA ΔpykA	[12]
Plasmids		
pTac15k	Km^R^, p15A origin, P_tac_ promoter, 4.0 kb	[13]
pTrc99A	Amp^R^, pBR322 origin, P_trc_ promoter, 4.2 kb	Pharmcia^b^
pYHP	pTac15k, P_tac_–aroG8/15–ydiB–aroK–pheA^fbr, dm,^ SacII region modification, P_pc113_-*glk*-T_lpp_, P_pc113_-*galP*-T_lpp_	[12]
pYHP-FDC	pYHP derivative, P_BBa_J23100_-*Sc*FDC (N-term 6×His-tag)	This study
pHB-CA	pTrc99A derivative, P_trc_–SmPAL (C-term FLAG-tag)	[12]
pHB-CA-FDC	pTrc99A derivative, P_trc_-SmPAL (C-term FLAG-tag)-ScFDC (N-term 6×His-tag)	This study

^a^Stratagene Cloning Systems, La Jolla, CA, USA

^b^Pharmacia biotech, Uppsala, Sweden

**Table 3:** Primer sequences used in this study

Primer	Sequence (5′ → 3′)
FDC-F	GCATGAATTCTTGAACTTTAAGAAGGAGATATACATATGCACCACCACCATCACCATAGGAAGCTAAATCCAGCTTTAGAATTTAGAG
FDC-R	ATGCTCTAGATTATCATTTATATCCGTACCTTTTCCAATTTTCATTTACTTTGTC
Gibson-F	CCGTTTACCGCTACTCTAGAGCGGCCGCTTGACGGCTAGCTCAGTCCTAGGTACAGTGCTAGCTTGAACTTTAAGAAGGAGATATACATATGCAC
Gibson-R1	GCACAATGTGCGCCATTTTTCACTTCACAGGTTTATCATTTATATCCGTACCTTTTCCAATTTTC
Gibson-R2	CAGGGAAGTGAGAGGGCCGCGGGCGGCCGCGTAGCGGTAAACGGCAGACAAAAAAAATGTCGCACAATGTGCGCCA

### Flask cultivation

Cells were inoculated in LB liquid medium (10 g/L tryptone, 5 g/L yeast extract, and 10 g/L NaCl) with 2% (w/v) glucose or PHE medium [13]. PHE medium composition is described below: 20 g/L glucose, 5 g/L (NH_4_)_2_SO_4_, 3 g/L KH_2_PO_4_, 3 g/L MgSO_4_·7H_2_O, 3 g/L yeast extract, 1.5 g/L sodium citrate, 1 g/L NaCl, 0.3 g/L l-tyrosine, 0.3 g/L l-tryptophan, 0.075 g/L thiamine-HCl, 0.015 g/L CaCl_2_·2H_2_O, 0.01125 g/L FeSO_4_·7H_2_O, and 1.5 mL/L Trace Metals Solution (TMS) at pH 6.8. TMS is composed of 15 g/L ZnSO_4_·7H_2_O, 14.64 g/L MnSO_4_·H_2_O, 12 g/L CaCO_3_, 3 g/L Na_2_MoO_4_·2H_2_O, 2.5 g/L NiSO_4_·6H_2_O, 2.5 g/L CuSO_4_·H_2_O, 2 g/L Al_2_(SO_4_)_3_·18H_2_O, 0.75 g/L CoSO_4_·7H_2_O, 0.5 g/L H_3_BO_3_, and 10 mL/L HCl. After overnight cultivation in LB or PHE medium, 2.5 mL of cells were transferred to 250 mL flasks containing 50 mL of fresh LB or PHE medium and 10 mL of *n*-dodecane. All flask cultivations were performed at 37 °C shaking at 200 rpm. Two antibiotics (100 μg/mL ampicillin and 40 μg/mL kanamycin) were added for plasmid maintenance, if necessary. When cells reached mid-exponential phase (OD_600_ of 1.0–1.2), IPTG (Sigma-Aldrich, St. Louis, MO, USA) was added to the final concentration of 1 mM to induce gene expression. After induction, cells were further cultivated in the same conditions (at 37 °C shaking with 200 rpm) which were previously optimized for higher production of precursors (l-phenylalanine and *t*CA) [12, 13].

### Fed-batch cultivation

Fed-batch cultivations were performed in a 5 L bioreactor (BioCNS, Daejeon, Republic of Korea). For seed culture (200 mL), cells were cultivated in PHE medium containing 3 g/L yeast extract at 37 °C and 200 rpm. After overnight cultivation, the seed was transferred into 1.8 L of same fresh medium in the bioreactor. Furthermore, 100 μg/mL ampicillin and 40 μg/mL kanamycin were also added for plasmid maintenance. An aerobic condition of 40% saturated dissolved oxygen (DO) concentration was maintained by automatically increasing the agitation speed up to 1000 rpm and by mixing pure oxygen during the cultivation [25]. The temperature was maintained at 37 °C, and the pH was kept at 6.8 by the automatic addition of 25% (v/v) ammonia solution, when the pH was lower than 6.77. When the pH value was greater than 6.86, an appropriate volume of feeding solution (500 g/L glucose, 100 g/L casamino acid, and 20 g/L MgSO_4_·7H_2_O) was automatically added to avoid glucose depletion. When cell density reached an OD_600_ of 45, IPTG was added to the final concentration of 1 mM. Moreover, 400 mL of *n*-dodecane was also added to the culture medium just before induction.

### Analytical procedures

In both flask and fed-batch cultivations, culture samples were periodically collected for the analysis of cell concentration and protein expression. Furthermore, to determine the styrene concentration during the cultivation, organic phases were acquired by phase separation via centrifugation (13,000 rpm for 10 min) of the culture broth. Styrene concentrations in the organic solvent (*n*-dodecane) was determined using gas chromatography (YL6500; YL instruments, Anyang, Korea) equipped with an HP-5 ms capillary column (30 m × 0.25 mm; Agilent Technology Inc., Santa Clara, CA, USA) [26]. After injection, the samples were detected and analyzed using a flame ionization detector (FID). Column temperature was set to increase from 40 to 135 °C at a rate of 20 °C/min. Styrene concentrations were determined by the standard curves.

## Additional files

**Additional file 1: Figure S1.** Schematic diagram of plasmid constructs for the expression of *Sc*FDC gene.

**Additional file 2: Figure S2.** SDS-PAGE analysis of genes expression.

**Additional file 3: Figure S3.** Effect of temperatures on styrene production.

**Additional file 4: Figure S4.** SDS-PAGE result of fed-batch cultivation with *n*-dodecane using *E. coli* YHP05 harboring pYHP-FDC and pHB-CA.
