# Supplementary material for: Enhanced production of styrene by engineered Escherichia coli and in situ product recovery (ISPR) with an organic solvent
Source: Microb Cell Fact. 2019 May 3;18:79. doi: 10.1186/s12934-019-1129-6 (PMC6498506; doi:10.1186/s12934-019-1129-6)
Supplement: Supplementary file 1 — Additional file 1: Figure S1. Schematic diagram of plasmid constructs for the expression of ScFDC gene. [file 12934_2019_1129_MOESM1_ESM.pdf]

A

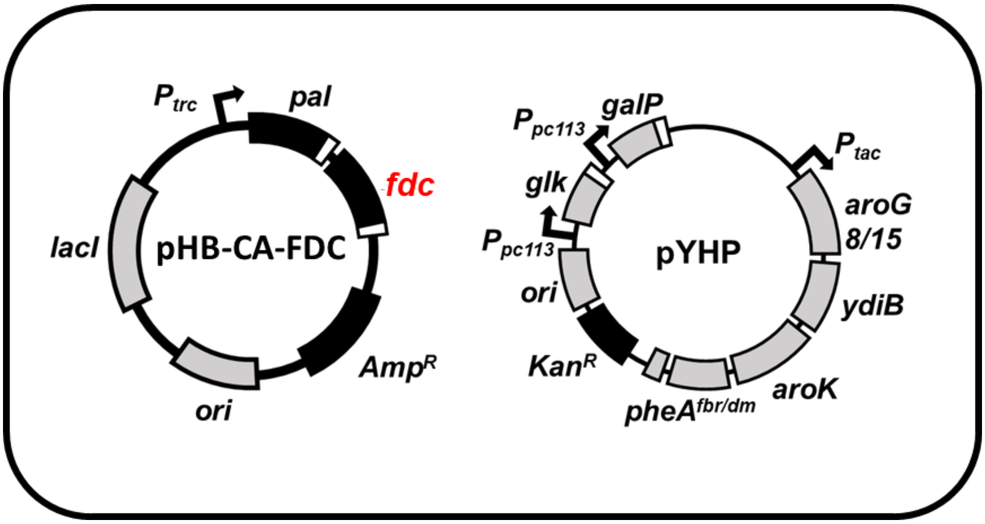

B

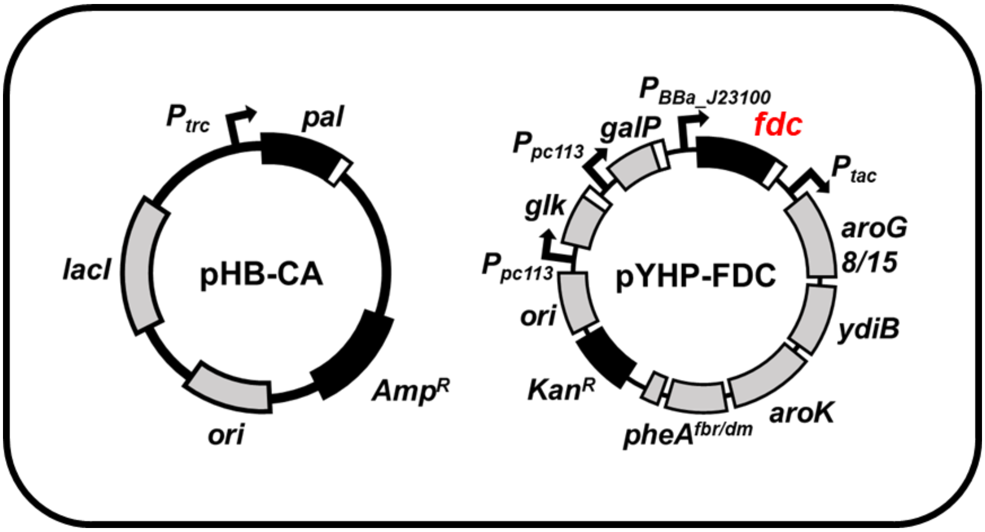

Additional file 1: Figure S1. Schematic diagram of plasmid constructs for the expression of *Sc*FDC gene. (A) *E. coli* YHP05 harboring pHB-CA-FDC and pYHP. ScFDC gene was cloned in pHB-CA-FDC under the P_{trc} promoter (B) *E. coli* YHP05 harboring pHB-CA and pYHP-FDC. ScFDC gene was cloned in pYHP-FDC under the constitutive BBa_J23100 promoter.
