# Supplementary material for: Enhanced production of styrene by engineered Escherichia coli and in situ product recovery (ISPR) with an organic solvent
Source: Microb Cell Fact. 2019 May 3;18:79. doi: 10.1186/s12934-019-1129-6 (PMC6498506; doi:10.1186/s12934-019-1129-6)
Supplement: Supplementary file 2 — Additional file 2: Figure S2. SDS-PAGE analysis of genes expression. [file 12934_2019_1129_MOESM2_ESM.pdf]

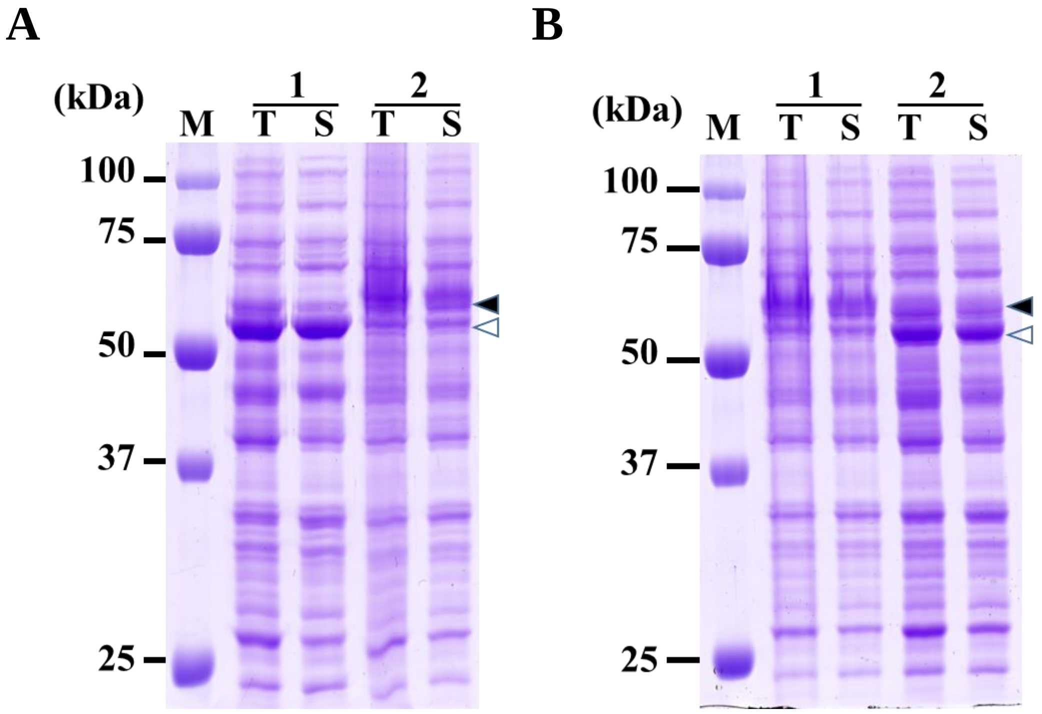

Additional file 2: Figure S2. SDS-PAGE analysis of genes expression (A) Protein samples of *E. coli* YHP05 harboring pYHP and pHB-CA (lane 1) and *E. coli* YHP05 harboring pYHP and pHB-CA-FDC (lane 2). (B) Protein samples of *E. coli* YHP05 harboring pYHP and pHB-CA –FDC (lane 1) and *E. coli* YHP05 harboring pYHP-FDC and pHB-CA (lane 2). Lanes M, T and S represent molecular weight markers (kDa), total and soluble fractions, respectively. Closed and open arrowheads indicate *Sc*FDC and *Sm*PAL enzymes, respectively.
