# Supplementary material for: Enhanced production of styrene by engineered Escherichia coli and in situ product recovery (ISPR) with an organic solvent
Source: Microb Cell Fact. 2019 May 3;18:79. doi: 10.1186/s12934-019-1129-6 (PMC6498506; doi:10.1186/s12934-019-1129-6)
Supplement: Supplementary file 3 — Additional file 3: Figure S3. Effect of temperatures on styrene production. [file 12934_2019_1129_MOESM3_ESM.pdf]

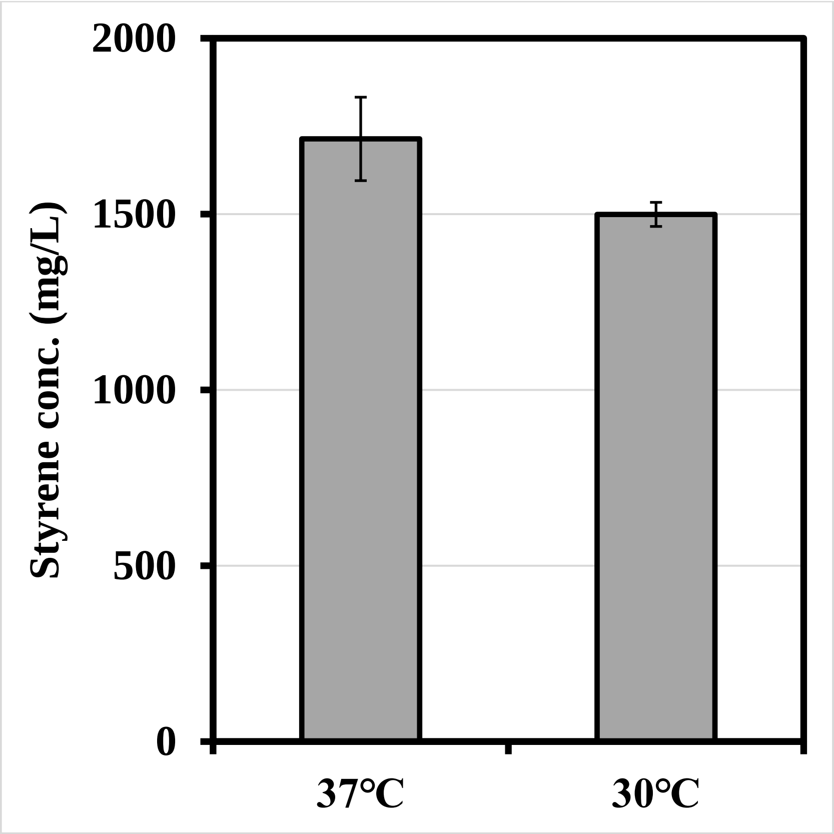

Additional file 3: Effect of temperatures on styrene production. *E. coli* YHP05 harboring pHB-CA and pYHP-FDC was cultivated in PHE medium containing yeast extract at 30°C and 37°C.
