# Supplementary material for: Enhanced production of styrene by engineered Escherichia coli and in situ product recovery (ISPR) with an organic solvent
Source: Microb Cell Fact. 2019 May 3;18:79. doi: 10.1186/s12934-019-1129-6 (PMC6498506; doi:10.1186/s12934-019-1129-6)
Supplement: Supplementary file 4 — Additional file 4: Figure S4. SDS-PAGE result of fed-batch cultivation with n-dodecane using E. coli YHP05 harboring pYHP-FDC and pHB-CA. [file 12934_2019_1129_MOESM4_ESM.pdf]

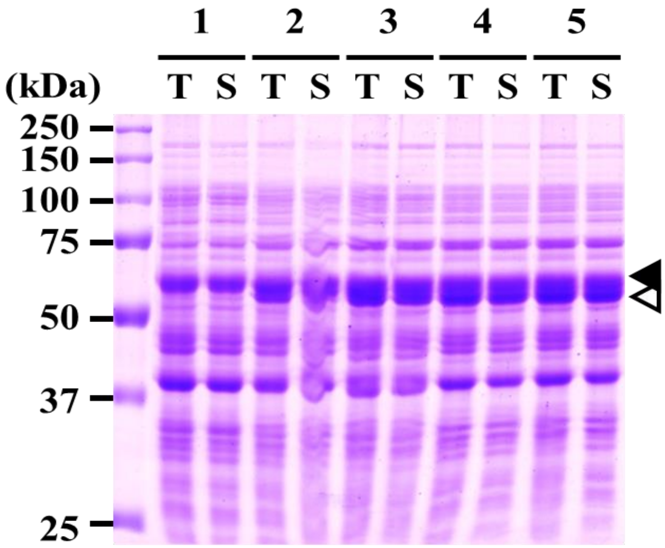

Additional file 4: Figure S4. SDS-PAGE result of fed-batch cultivation with n-dodecane using *E. coli* YHP05 harboring pYHP-FDC and pHB-CA. Lanes 1-5 represent the samples at 0 h, 8.5 h, 14 h, 23 h, 37 h after induction. Lanes T and S represent total and soluble fractions, respectively. Closed and open arrowheads indicate *Sm*PAL and *Sc*FDC enzymes, respectively.
